# Analysis of the relationship between physical education majors’ perceptions of their body shape and fatphobia

**DOI:** 10.3389/fpsyg.2025.1709560

**Published:** 2026-01-12

**Authors:** Canan Sayin Temur, Mustafa Can Koc, Teodora Mihaela Iconomescu, Laurentiu-Gabriel Talaghir

**Affiliations:** 1 Faculty of Sport Sciences, Ankara Yildirim Beyazit University, Ankara, Türkiye; 2 Faculty of Sport Sciences, Istanbul Gelisim University, Istanbul, Türkiye; 3 Faculty of Physical Education and Sport, Dunarea de Jos University of Galati, Galati, Romania

**Keywords:** fatphobia, body dissatisfaction, PE, teacher, candidates

## Abstract

**Purpose:**

The purpose of this study is to investigate whether body shape perceptions and fat phobia differ according to the gender and year of study of PE teacher candidates. In addition, the study aims to investigate the possible relationship between actual and ideal body shape perceptions and fatphobia.

**Methods:**

The study involved a total of 141 PE teacher candidates. The participants’ mean age was 21.45 (±1.93) years (M female = 21.08 ± 1.56, M male = 21.73 ± 2.14). Data was collected using a Demo-graphic Information Form, a Fatphobia Scale, and a Figure Rating Scale. The Mann–Whitney U test, the Kruskal–Wallis test and the Spearman’s correlation analysis were used to analyze the data.

**Results:**

The results showed that there was no statistically significant difference between female and male teacher candidates’ levels of fatphobia. In contrast, there was a significant difference between their perceptions of current and ideal body shape. The results also showed that there was no difference between the perceived levels of fatphobia and the perceived current and ideal body shapes of the Grade Level 1st, 2nd, 3rd, and 4th-year PE teacher candidates. The findings revealed a lack of a statistically significant relationship between fatphobia and current body image and between fatphobia and ideal body image.

**Conclusion:**

As a result of the research, the fact that PE teacher candidates are moderately fatphobic, perceive themselves to be overweight, and idealize thinness to a higher degree than men has provided critical insights for educational policymakers.

## 1 Introduction

In recent years, there has been a substantial surge in scholarly inquiry focused on body dissatisfaction. This surge aligns with the notable increase in levels of body dis-satisfaction observed among children, adolescents, and adults. This trend has prompted heightened academic and clinical attention to the complex factors contributing to and exacerbating body dissatisfaction across various age groups (Smolak, 2004). According to the findings of these studies, it has been revealed that a significant proportion of children and adolescents are dissatisfied with their physical appearance (Bucchianeri et al., 2013; Grogan, 2021). ‘Studies in the literature have made the important observation that individuals’ dissatisfaction with their bodies is low in the period before starting school’ (Birbeck and Drummond, 2005; Cramer and Steinwert, 1998; McCabe et al., 2007; Pallan et al., 2011; Tatangelo et al., 2016). Research has shown that body dissatisfaction experienced during childhood and adolescence has long-lasting effects that extend into adulthood. This dissatisfaction can lead to a variety of issues, including an increased risk of developing eating disorders, depression, and anxiety. The impacts of these early experiences on body image can have profound implications for mental and physical well-being later in life (Kokka et al., 2023; Pulgarón, 2013).

Body dissatisfaction is a common issue in today’s society and is often exacerbated by external pressures, particularly from the media. The portrayal of unrealistic standards of beauty in the media can have detrimental effects by perpetuating unattainable ideals and perpetuating stereotypes. This can negatively affect individuals, impacting their mental well-being and contributing to dissatisfaction and insecurity (Scully et al., 2020; Vuong et al., 2021). The negative feelings, thoughts, and behaviors directed toward individuals with larger body size, stemming from societal pressures to conform to an ideal of thinness, have farreaching destructive effects. Not only do these attitudes stigmatize, discriminate against, and exclude individuals who do not fit the idealized body size, but they also contribute to a culture that can be damaging to mental and physical well-being (Arroyo, 2014; Kite et al., 2022; Rubino et al., 2020). This situation leads to an increase in the problems of individuals with in-ternal problems based on body dissatisfaction due to external factors (Bucchianeri and Neumark-Sztainer, 2014; Puhl et al., 2017; Latzer and Stein, 2013).

Research consistently shows a strong link between higher body weight and body dissatisfaction (Duncan et al., 2006; Stice and Shaw, 2002; Martins et al., 2010). Negative stereotypes and attitudes toward overweight individuals, known as fatphobia, significantly contribute to this dissatisfaction by shaping how individuals perceive and feel about their bodies (Bacon et al., 2001). Fatphobia’s harmful effects extend across various social domains, including employment, healthcare, and family life (Robinson et al., 1993; Puhl and Brownell, 2003). Schools are also common settings for weight-based discrimination, where ridicule and negative behaviors can severely affect students’ well-being and self-esteem (Griffiths et al., 2005; Eisenberg et al., 2003; Clare et al., 2014; Puhl et al., 2011). One study found that overweight high school students were the main targets of teasing, with 84% of participants reporting they had witnessed such ridicule during physical activities (Puhl et al., 2011).

The existing body of literature studying body shape concerns, and fatphobia emphasizes the urgent need for further research, especially in sports science (Richardson et al., 2019). It is imperative to conduct a comprehensive examination of body shape concerns and fatphobia in prospective PE teachers. These educators play a pivotal role in shaping positive body image messages. They must avoid contributing to body dissatisfaction or body image anxiety in the children and young people they will be instructing (Quennerstedt et al., 2021; Varea and Underwood, 2016). Prevention of the internalization of body dissatisfaction and fatphobia from an early age in children and young people is crucial, as it aims to deter the development of negative attitudes and behaviors associated with these issues (Cliff and Wright, 2010; Wrench and Garrett, 2013). The insights gleaned from these studies will be instrumental in deepening our understanding of body dissatisfaction and fatphobia, specifically among PE teacher candidates. By examining body shape perceptions and fatphobia, we aim to explore potential differences based on the PE teacher candidates gender and year of study. Through this study, we strive to uncover any possible correlations between body shape perceptions and fatphobia, which will ultimately enable us to effectively address and support the diverse needs of individuals within the PE and sports environment. Our goal is to create a more inclusive and supportive setting that caters to the holistic well-being of all individuals involved. For these reasons, the purpose of this study is to examine if body shape perceptions and fat phobia differ based on the gender and year of study of PE teacher candidates. Additionally, the study examines the potential relationship between actual and ideal body shape perceptions and fatphobia.

## 2 Methods

### 2.1 Research design

In this research, we conducted a correlational descriptive study utilizing a cross-sectional survey design to investigate the variations in levels of fatphobia and perceptions of actual and ideal body shape among PE teacher candidates based on gender and grade level (Karasar, 2005).

### 2.2 Universe and sample

A total of 141 candidates PE teacher studying in the Department of Physical Education and Sport Education took part in the study. The participants’ mean age was 21.45 (±1.93) years (M female = 21.08 ± 1.56, M male = 21.73 ± 2.14). The demographics of those participating are shown in Table 1.

**Table 1:** Demographic information of the participants.

Variables	*n*	*%*
Gender		
Female	62	44.0
Male	79	56.0
Total	141	100
Grade level		
1st grade	33	23.4
2nd grade	38	27.0
3rd grade	35	24.8
4th grade	35	24.8
Total	141	100

### 2.3 Data collection tools

Data were collected using a Demographic Information Form, a Fat Phobia Scale, and a Figure Rating Scale.

#### 2.3.1 Demographic Information Form

The Demographic Information Form included questions about the participants’ gender, grade level, and age.

#### 2.3.2 Fat Phobia Scale

To determine PE teachers’ beliefs and feelings toward overweight or obese individuals, the Fat Phobia Scale developed by Bacon et al. (2001) and adapted to Turkish culture by Koçak et al. (2005) was used. The fatphobia scale contains a total of 14 pairs of contrasting adjectives, such as “overeats - undereats,” and participants are asked to indicate their opinion of obese people on a scale of 1–5 between the pairs, closest to the adjective they feel best describes their feelings toward overweight people. To calculate the total score on the Fatphobia Scale, you need to add up the assigned values for each adjective and then divide the sum by 14. The scores for fatphobia can range from 1 to 5. A score closer to 5 indicates a high level of fatphobia, while a score closer to 1 indicates a low level of fatphobia. The Cronbach’s alpha value calculated when developing the scale was 0.91, and the Cronbach’s alpha value calculated when adapting the scale to Turkish culture was 0.82. In this study, the reliability coefficient of the scale was calculated to be 0.85.

#### 2.3.3 Figure Rating Scale

The Stunkard Figure Rating Scale (SFRS), developed by Stunkard et al. (1983), was used as a data collection tool to determine the current and ideal body shape perceptions of the candidate PE teachers in the study. The SFRS includes 9 body shapes, from extremely thin to obese, representing both females and males. The participants were first asked to identify the shape that represented their current body shape from among the body shapes numbered from 1 to 9 in the SFRS and then to determine the ideal body shape that they considered themselves to have. The SFRS was found to be a reliable tool for collecting data from university and PE students (Hamurcu, 2023; Langdon et al., 2016).

### 2.4 Data analysis

The data collection procedure began with obtaining approval from the university’s ethics committee where the data were being gathered and official permission from the institute where the data were being gathered. Appropriate courses for the students were identified, and permission to administer the data collection instruments was obtained from the course lecturers following the approval and permission processes. Students in the courses for which permission had been obtained were informed of the research, and the data collection tools were only made available to students who were volunteers for the study. It took 10 min for the students to fill in the data collection tools.

Due to the non-normal distribution of the research data, the Mann–Whitney U test was used to determine whether there was a difference between the levels of fatphobia and current and ideal body shape perceptions of PE teacher candidates according to their gender, and the Kruskal–Wallis test to determine whether there was a difference according to grade level, and Spearman’s correlation analysis to determine whether there was a relationship between fatphobia and current and ideal body shape perceptions. The data was analyzed using IBM SPSS Statistics for Windows version 21.0.

## 3 Results

This section contains the table and comments resulting from the analysis performed.

The Mann–Whitney U test was conducted to examine gender differences in fatphobia levels, and current and ideal body shape perceptions among pre-service PE teachers. The analysis indicated that there was no significant difference in fatphobia levels between female (Med = 2.93) and male (Med = 2.86) participants, U = 2170.00, z = −1.160, *p* = 0.246. However, significant differences were observed in body shape perceptions. Males reported higher scores than females for both current body shape (MedFemale = 4.00, MedMale = 4.00, U = 1407.50, z = −4.468, *p* < 0.001) and ideal body shape (MedFemale = 3.00, MedMale = 4.00, U = 898.50, z = −6.740, p < 0.001), indicating gender-specific differences in body image perceptions (Table 2).

**Table 2:** Mann–Whitney *U* test results for fatphobia, perceived actual and ideal body shape of female and male participants.

Gender		*n*	*x̄*	*SD*	Med.	*U*
Fatphobia	Female	62	2.97	0.67	2.93	2170.00
	Male	79	2.85	0.76	2.86	
Perceived actual body shape	Female	62	3.37	0.98	4.00	1407.50*
	Male	79	4.31	1.23	4.00	
Perceived ideal body shape	Female	62	3.15	0.74	3.00	898.50*
	Male	79	4.20	0.85	4.00	

*p* < 0.001.

The Kruskal–Wallis test was used to determine whether there was a statistically significant difference between the levels of fatphobia, and current and ideal body shape perceptions of PE teacher candidates in the 1st, 2nd, 3rd, and 4th years of study. The results of the analysis revealed that there was no statistically significant difference between the perceptions of the 1st, 2nd, 3rd, and 4th-grade candidate PE teachers about their levels of fatphobia [H(3) = 2.89, *p* = 0.409], current [H(3) = 3.25, *p* = 0.355] and ideal [H(3) = 4.13, *p* = 0.248] body shape (Table 3).

**Table 3:** Kruskal–Wallis *H* test results for fatphobia, perceived actual and ideal body shape of 1st-, 2nd-, 3rd-, and 4th- graders.

Grade		*n*	*x̄*	*SD*	Mean Rank	*H*
Fatphobia	1st grade	33	3.08	0.88	80.42	2.89
	2nd grade	38	2.77	0.65	64.09	
	3rd grade	35	2.87	0.59	69.63	
	4th grade	35	2.92	0.74	70.99	
Perceived actual body shape	1st grade	33	4.03	1.07	75.36	3.25
	2nd grade	38	3.61	1.13	62.16	
	3rd grade	35	4.09	1.40	77.43	
	4th grade	35	3.89	1.23	70.06	
Perceived ideal body shape	1st grade	33	3.97	0.77	79.82	4.13
	2nd grade	38	3.58	0.92	64.18	
	3rd grade	35	3.83	1.12	75.81	
	4th grade	35	3.60	0.98	65.27	

As there was a difference between levels of fatphobia and perceived current and ideal body shape according to the gender of the candidate teachers who participated in the study and no difference according to the grade level they taught, the data on the relationship between fatphobia and perceived current and ideal body shape were analyzed separately by gender. Spearman’s correlation analysis was used to determine whether there was a statistically significant relationship between the fatphobia of female and male candidate PE teacher candidates and their perceived current and ideal body shape. The results of the analysis showed that there was no statistically significant correlation between the fatphobia of female and male candidate PE teachers and their perceived current body shape [rsfemale = 0.005, *p* = 0.970; rsmale = 0.159, *p* = 0.162] and between fatphobia and ideal body shape [rsfemale = 0.135, *p* = 0.294; rsmale = 0.211, *p* = 0.061] (Table 4).

**Table 4:** Correlations between fatphobia and actual and ideal body shape perceptions of female and male PE teacher candidates.

Female			
		Perceived actual body shape	Perceived ideal body shape
Fatphobia	*rs*	0.005	0.135
	*p*	0.970	0.294
	*N*	62	62

Male			
		Perceived actual body shape	Perceived ideal body shape l
Fatphobia	*rs*	0.159	0.211
	*p*	0.162	0.061
	*N*	79	79

## 4 Discussion

In this study, the levels of fatphobia among pre-service physical education (PE) teachers were examined in relation to gender and grade level. The findings revealed that fatphobia levels did not differ significantly across these variables, and that, overall, participants exhibited moderate levels of fatphobia.

Previous research in this area has yielded mixed results. Some studies have reported no gender differences in fatphobia (Fidancı et al., 2021; Zaroubi et al., 2021), whereas others have identified higher levels among women (Tapking et al., 2020; Hayran et al., 2013; Ayhan Başer et al., 2021). For example, studies involving health professionals in Germany found that women used more positive descriptors for individuals with obesity, suggesting lower levels of fatphobia (Tapking et al., 2020). Similarly, research involving exercise science students and sports coaches also reported moderate levels of fatphobia with no gender-based differences (Langdon et al., 2016; Winter et al., 2024; Elboim-Gabyzon et al., 2020). These findings align with the present study, which also identified moderate and comparable levels of fatphobia among male and female pre-service PE teachers.

The similarity in fatphobia levels across genders may be attributed to the participants’ shared experiences and professional background. Both male and female candidates were admitted to PE programs through aptitude examinations and likely possess similar levels of exposure to sport and body related norms. Consequently, they may experience comparable societal and professional pressures to maintain an athletic and fit appearance, resulting in similar attitudes toward obesity.

The results concerning body shape perception revealed that female pre-service PE teachers viewed both their current and ideal body shapes as slimmer than those of male participants. This finding is consistent with existing literature demonstrating that women tend to experience greater body dissatisfaction and a stronger preference for thinness than men (Gruszka et al., 2022; O’Dea, 1999; Olatona et al., 2023; Radwan et al., 2019). In contrast, men are more likely to idealize a muscular physique. Such gender-specific body image perceptions have been observed across diverse cultural contexts, including studies conducted in Poland, Australia, Nigeria, and the United Arab Emirates. These differences are often attributed to the greater societal pressure placed on women to conform to thinness ideals (Swami, 2015; Kiefer et al., 2000; Barker et al., 2021).

Although participants in this study were likely to be physically active and fit, the observed discrepancy between perceived and ideal body shapes particularly among female participants indicates internalized societal expectations. The emphasis on thinness and fitness among PE teachers may further reinforce these perceptions, contributing to moderate levels of body dissatisfaction even among physically active individuals.

The study also found no significant relationship between fatphobia and perceived actual or ideal body shape. This suggests that while participants held moderate fatphobic attitudes, these were not directly linked to their personal body image perceptions. Similar findings have been reported among fitness center users and exercise science students, where moderate fatphobia coexisted with strong athletic body ideals (Olatona et al., 2023; Eroğlu and Eroğlu, 2020).

Research has shown that PE teachers often associate body weight with ability, competence, and motivation (Varea and Underwood, 2016; Greenleaf and Weiller, 2005). Such attitudes may inadvertently reinforce weight stigma and hinder the development of inclusive physical activity environments. The current findings underscore the importance of addressing these biases within PE teacher education programs, emphasizing awareness of weight stigma and promoting a more holistic understanding of health and physical competence. In conclusion, this study highlights the moderate yet consistent presence of fatphobia among pre-service PE teachers, with no significant gender or grade level differences. The results emphasize the need for educational interventions that challenge weight-based stereotypes and support the development of equitable attitudes toward individuals of all body sizes within the context of physical education and sports.

## 5 Conclusion

This study examined fatphobia and body shape perceptions among pre-service physical education teachers in relation to gender and year of study. The results showed that participants exhibited moderate levels of fatphobia, with no significant differences across gender or grade levels. Moreover, fatphobic attitudes were not associated with perceptions of actual or ideal body shape.

These findings indicate that pre-service PE teachers, regardless of gender, share similar attitudes toward body weight and shape. The presence of moderate fatphobia highlights the need to address weight-related biases within PE teacher education programs to foster more inclusive perspectives in future professional practice.

Future research could explore the sociocultural and educational factors influencing these attitudes through mixed-method designs, extending the current findings to broader contexts within physical education and sport.

## Data Availability

The data analyzed in this study is subject to the following licenses/restrictions: it has been preferred to share it with permission to prevent data theft and within the scope of protecting personal data. Requests to access these datasets should be directed to the first author, CS, canansayintemur@aybu.edu.tr.
